# 4-Methyl­anilinium perchlorate 18-crown-6 clathrate

**DOI:** 10.1107/S1600536811053992

**Published:** 2011-12-21

**Authors:** Yu-Feng Wang

**Affiliations:** a Ordered Matter Science Research Center, Southeast University, Nanjing 211189, People’s Republic of China

## Abstract

In the title compound, C_7_H_10_N^+^·ClO_4_^−^·C_12_H_24_O_6_, the 4-methyl­anilinium cation inter­acts with an 18-crown-6 mol­ecule forming a rotator–stator-like structure through bifurcated N—H⋯(O,O) hydrogen bonds between the ammonium group of the cation and the O atoms of the crown ether mol­ecule. All three components of the structure possess mirror symmetry. The benzene ring is inclined to the mean plane of the crown ether molecule by 86.84 (8)°.

## Related literature

The crystal structure of related 4-methyl­anilinium tetra­fluoro­borate 18-crown-6 clathrate has been reported by Ge & Zhao (2010 ▶).
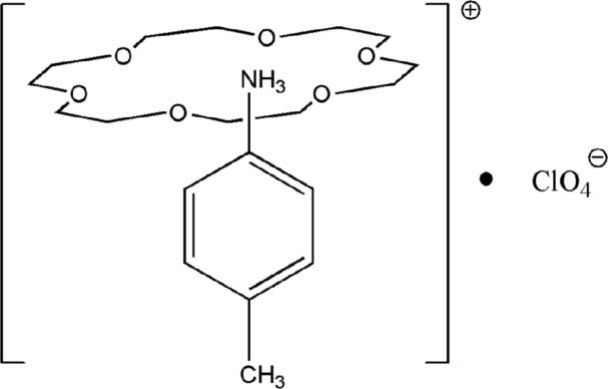


## Experimental

### 

#### Crystal data

C_7_H_10_N^+^·ClO_4_^−^·C_12_H_24_O_6_
*M* *_r_* = 471.92
Orthorhombic, 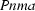

*a* = 15.510 (3) Å
*b* = 11.717 (2) Å
*c* = 13.014 (3) Å
*V* = 2365.0 (8) Å^3^
*Z* = 4
Mo *K*α radiation
μ = 0.21 mm^−1^
*T* = 293 K
0.27 × 0.26 × 0.23 mm

#### Data collection

Rigaku Mercury2 diffractometer
Absorption correction: multi-scan (*CrystalClear*; Rigaku, 2005 ▶) *T*_min_ = 0.944, *T*_max_ = 0.952
23471 measured reflections
2843 independent reflections
2051 reflections with *I* > 2σ(*I*)
*R*_int_ = 0.056

#### Refinement

*R*[*F* ^2^ > 2σ(*F* ^2^)] = 0.052
*wR*(*F* ^2^) = 0.131
*S* = 1.04
2843 reflections
156 parameters
H-atom parameters constrained
Δρ_max_ = 0.23 e Å^−3^
Δρ_min_ = −0.29 e Å^−3^

### 

Data collection: *CrystalClear* (Rigaku, 2005 ▶); cell refinement: *CrystalClear*; data reduction: *CrystalClear*; program(s) used to solve structure: *SHELXS97* (Sheldrick, 2008 ▶); program(s) used to refine structure: *SHELXL97* (Sheldrick, 2008 ▶); molecular graphics: *SHELXTL* (Sheldrick, 2008 ▶); software used to prepare material for publication: *SHELXTL*.

## Supplementary Material

Crystal structure: contains datablock(s) I, global. DOI: 10.1107/S1600536811053992/cv5196sup1.cif

Structure factors: contains datablock(s) I. DOI: 10.1107/S1600536811053992/cv5196Isup2.hkl

Supplementary material file. DOI: 10.1107/S1600536811053992/cv5196Isup3.cml

Additional supplementary materials:  crystallographic information; 3D view; checkCIF report

## Figures and Tables

**Table 1:** Hydrogen-bond geometry (Å, °)

*D*—H⋯*A*	*D*—H	H⋯*A*	*D*⋯*A*	*D*—H⋯*A*
N1—H1*B*⋯O2^i^	0.89	2.23	2.9477 (19)	138
N1—H1*B*⋯O3^i^	0.89	2.19	2.9511 (18)	143
N1—H1*C*⋯O4	0.89	2.20	2.924 (3)	138
N1—H1*C*⋯O3	0.89	2.20	2.9511 (18)	142
N1—H1*A*⋯O2	0.89	2.22	2.9477 (19)	139
N1—H1*A*⋯O1	0.89	2.15	2.888 (3)	140

Symmetry code: (i) 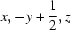.

**Fig. 1.**
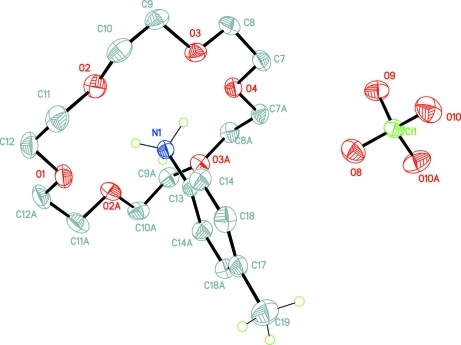
The molecular structure of the title compound, showing the atomic numbering scheme with 30% probability displacement ellipsoids [symmetry code: (A) x, 1/2-y, z].

## supplementary crystallographic information

### Comment

Recently, the crystal structure of 4-methylanilinium tetrafluoroborate 18-crown-6 clathrate (I), obtained in our laboratory, has been reported (Ge & Zhao, 2010). In continuation of our studies of compounds containing 18-crown-6 macrocycles and ammonium cations RNH_3_^+^, we present here the title compound (II) (Fig. 1), which is isostructural with (I).

In (II), the methyl and the protonated –NH_3_ groups of the 4-methylanilinium lie on a dual axis of rotation, and perchlorate anion lie on a mirror plane. All bond length and angles are normal and correspond to those reported for (I) (Ge & Zhao, 2010). The 4-methylanilinium cation interacts with 18-crown-6 molecule forming a rotator–stator structure through bifurcated N—H···(O,*O*) hydrogen bonds (Table 1) between the ammonium group of the cation and the O atoms of the crown ether molecule.

### Experimental

In room temperature *p*-tolylanmine (5 mmol, 0.535 g) was dissolved in 20 ml Me thanol, then HClO_4_ was added into the previous solution slowly with properly sirring until the solution become neutral.At last 18-crown-6 (5 mmol,1.65 g) were dissolved in the solution above, strring. Quite a quantity of white deposit are obtained,methanol are added into the solution again until deposit are dissolved. A great quantity of colorless block crystasls were obtained by filtrating after several hours in air. Block colorless single crystals suitable for X-ray structure analysis were obtained by the slow evaporation of the above solution after a week in air.

The dielectric constant of the compound as a function of temperature indicates that the permittivity is basically temperature-independent (ε = C/(T–T_0_)), suggesting that this compound is not ferroelectric or there may be no distinct phase transition occurring within the measured temperature range between 128 K and 378 K.

### Refinement

H atoms were placed in calculated positions (C—H = 0.93–0.97 Å; N—H = 0.89 Å) and refined as riding, with *U*_iso_ = 1.2-1.5 *U*eq of the parent atom.

### Crystal data

**Table:**

C_7_H_10_N^+^·ClO_4_^−^·C_12_H_24_O_6_	*F*(000) = 1008
*M**_r_* = 471.92	*D*_x_ = 1.325 Mg m^−^^3^
Orthorhombic, *P**n**m**a*	Mo *K*α radiation, λ = 0.71073 Å
Hall symbol: -P 2ac 2n	θ = 3.1–27.8°
*a* = 15.510 (3) Å	µ = 0.21 mm^−^^1^
*b* = 11.717 (2) Å	*T* = 293 K
*c* = 13.014 (3) Å	Block, colourless
*V* = 2365.0 (8) Å^3^	0.27 × 0.26 × 0.23 mm
*Z* = 4	

### Data collection

**Table:**

Rigaku Mercury2 diffractometer	2843 independent reflections
Radiation source: fine-focus sealed tube	2051 reflections with *I* > 2σ(*I*)
graphite	*R*_int_ = 0.056
Detector resolution: 13.6612 pixels mm^-1^	θ_max_ = 27.5°, θ_min_ = 3.1°
CCD_Profile_fitting scans	*h* = −20→19
Absorption correction: multi-scan (*CrystalClear*; Rigaku, 2005)	*k* = −15→15
*T*_min_ = 0.944, *T*_max_ = 0.952	*l* = −16→16
23471 measured reflections	

### Refinement

**Table:**

Refinement on *F*^2^	Primary atom site location: structure-invariant direct methods
Least-squares matrix: full	Secondary atom site location: difference Fourier map
*R*[*F*^2^ > 2σ(*F*^2^)] = 0.052	Hydrogen site location: inferred from neighbouring sites
*wR*(*F*^2^) = 0.131	H-atom parameters constrained
*S* = 1.04	*w* = 1/[σ^2^(*F*_o_^2^) + (0.0508*P*)^2^ + 0.9055*P*] where *P* = (*F*_o_^2^ + 2*F*_c_^2^)/3
2843 reflections	(Δ/σ)_max_ < 0.001
156 parameters	Δρ_max_ = 0.23 e Å^−^^3^
0 restraints	Δρ_min_ = −0.29 e Å^−^^3^

### Special details

**Table:**

Geometry. All e.s.d.'s (except the e.s.d. in the dihedral angle between two l.s. planes) are estimated using the full covariance matrix. The cell e.s.d.'s are taken into account individually in the estimation of e.s.d.'s in distances, angles and torsion angles; correlations between e.s.d.'s in cell parameters are only used when they are defined by crystal symmetry. An approximate (isotropic) treatment of cell e.s.d.'s is used for estimating e.s.d.'s involving l.s. planes.
Refinement. Refinement of *F*^2^ against ALL reflections. The weighted *R*-factor *wR* and goodness of fit *S* are based on *F*^2^, conventional *R*-factors *R* are based on *F*, with *F* set to zero for negative *F*^2^. The threshold expression of *F*^2^ > σ(*F*^2^) is used only for calculating *R*-factors(gt) *etc*. and is not relevant to the choice of reflections for refinement. *R*-factors based on *F*^2^ are statistically about twice as large as those based on *F*, and *R*- factors based on ALL data will be even larger.

### Fractional atomic coordinates and isotropic or equivalent isotropic displacement parameters (Å^2^)

**Table:**

	*x*	*y*	*z*	*U*_iso_*/*U*_eq_	Occ. (<1)
Cl1	0.39923 (4)	0.2500	0.29294 (6)	0.0499 (2)	
O9	0.48479 (14)	0.2500	0.33632 (19)	0.0668 (6)	
O10	0.38749 (12)	0.14964 (16)	0.23197 (15)	0.0872 (6)	
O8	0.33818 (15)	0.2500	0.37618 (19)	0.0764 (7)	
O3	0.39105 (9)	0.03863 (11)	0.71904 (11)	0.0521 (4)	
O4	0.45688 (13)	0.2500	0.65171 (14)	0.0500 (5)	
N1	0.29743 (14)	0.2500	0.77156 (16)	0.0419 (5)	
H1A	0.2845	0.2166	0.8310	0.063*	0.50
H1B	0.3141	0.3216	0.7830	0.063*	0.50
H1C	0.3400	0.2118	0.7412	0.063*	0.50
O2	0.29941 (9)	0.04793 (12)	0.90648 (11)	0.0584 (4)	
O1	0.21558 (14)	0.2500	0.97086 (15)	0.0608 (6)	
C13	0.22121 (16)	0.2500	0.70472 (18)	0.0392 (6)	
C9	0.39753 (15)	−0.04759 (18)	0.79645 (17)	0.0588 (6)	
H9A	0.4433	−0.0283	0.8442	0.071*	
H9B	0.4114	−0.1203	0.7649	0.071*	
C8	0.46909 (14)	0.04990 (18)	0.66202 (17)	0.0564 (5)	
H8A	0.4795	−0.0190	0.6227	0.068*	
H8B	0.5171	0.0608	0.7087	0.068*	
C14	0.18536 (14)	0.14842 (18)	0.67484 (15)	0.0533 (5)	
H14	0.2097	0.0797	0.6956	0.064*	
C7	0.46218 (15)	0.14939 (18)	0.59148 (15)	0.0545 (5)	
H7A	0.5123	0.1528	0.5470	0.065*	
H7B	0.4112	0.1421	0.5488	0.065*	
C10	0.31436 (15)	−0.05658 (18)	0.85247 (18)	0.0611 (6)	
H10A	0.2678	−0.0705	0.8043	0.073*	
H10B	0.3165	−0.1197	0.9006	0.073*	
C11	0.22117 (15)	0.0464 (2)	0.9626 (2)	0.0705 (7)	
H11A	0.2175	−0.0228	1.0032	0.085*	
H11B	0.1726	0.0482	0.9158	0.085*	
C12	0.21869 (16)	0.1486 (2)	1.03166 (17)	0.0720 (7)	
H12A	0.1683	0.1448	1.0757	0.086*	
H12B	0.2696	0.1497	1.0750	0.086*	
C17	0.07397 (19)	0.2500	0.5819 (2)	0.0558 (8)	
C18	0.11245 (14)	0.1491 (2)	0.61334 (16)	0.0598 (6)	
H18	0.0887	0.0799	0.5926	0.072*	
C19	−0.0071 (2)	0.2500	0.5175 (3)	0.0878 (12)	
H19A	−0.0486	0.1992	0.5474	0.132*	0.50
H19B	0.0064	0.2250	0.4491	0.132*	0.50
H19C	−0.0305	0.3258	0.5150	0.132*	0.50

### Atomic displacement parameters (Å^2^)

**Table:**

	*U*^11^	*U*^22^	*U*^33^	*U*^12^	*U*^13^	*U*^23^
Cl1	0.0481 (4)	0.0428 (4)	0.0587 (4)	0.000	0.0021 (3)	0.000
O9	0.0551 (13)	0.0592 (13)	0.0862 (16)	0.000	−0.0069 (12)	0.000
O10	0.0857 (13)	0.0753 (12)	0.1007 (13)	−0.0023 (10)	−0.0109 (11)	−0.0348 (11)
O8	0.0657 (15)	0.0856 (17)	0.0778 (16)	0.000	0.0166 (13)	0.000
O3	0.0547 (8)	0.0424 (7)	0.0593 (9)	0.0062 (6)	0.0008 (7)	0.0064 (6)
O4	0.0629 (12)	0.0435 (11)	0.0437 (10)	0.000	0.0081 (9)	0.000
N1	0.0443 (12)	0.0426 (12)	0.0388 (12)	0.000	0.0047 (10)	0.000
O2	0.0560 (9)	0.0527 (9)	0.0664 (9)	−0.0097 (7)	0.0087 (7)	0.0088 (7)
O1	0.0650 (13)	0.0781 (15)	0.0393 (11)	0.000	0.0077 (10)	0.000
C13	0.0397 (13)	0.0461 (14)	0.0319 (12)	0.000	0.0051 (11)	0.000
C9	0.0688 (14)	0.0412 (11)	0.0665 (14)	0.0059 (10)	−0.0085 (12)	0.0068 (10)
C8	0.0589 (12)	0.0497 (12)	0.0605 (13)	0.0122 (10)	0.0065 (11)	−0.0074 (10)
C14	0.0597 (12)	0.0468 (12)	0.0534 (12)	−0.0010 (10)	−0.0048 (10)	0.0000 (9)
C7	0.0610 (12)	0.0549 (13)	0.0477 (11)	0.0044 (10)	0.0069 (10)	−0.0079 (10)
C10	0.0699 (14)	0.0433 (12)	0.0700 (14)	−0.0104 (10)	−0.0115 (12)	0.0142 (10)
C11	0.0611 (14)	0.0783 (17)	0.0721 (16)	−0.0125 (12)	0.0103 (12)	0.0237 (14)
C12	0.0649 (14)	0.106 (2)	0.0453 (12)	−0.0048 (14)	0.0128 (11)	0.0184 (14)
C17	0.0428 (15)	0.082 (2)	0.0421 (15)	0.000	0.0020 (13)	0.000
C18	0.0607 (13)	0.0635 (14)	0.0550 (12)	−0.0145 (11)	−0.0021 (11)	−0.0045 (11)
C19	0.060 (2)	0.126 (4)	0.077 (3)	0.000	−0.016 (2)	0.000

### Geometric parameters (Å, °)

**Table:**

Cl1—O10	1.4302 (17)	C8—C7	1.488 (3)
Cl1—O10^i^	1.4302 (17)	C8—H8A	0.9700
Cl1—O8	1.439 (2)	C8—H8B	0.9700
Cl1—O9	1.442 (2)	C14—C18	1.385 (3)
O3—C8	1.426 (2)	C14—H14	0.9300
O3—C9	1.430 (2)	C7—H7A	0.9700
O4—C7^i^	1.418 (2)	C7—H7B	0.9700
O4—C7	1.418 (2)	C10—H10A	0.9700
N1—C13	1.468 (3)	C10—H10B	0.9700
N1—H1A	0.8900	C11—C12	1.497 (3)
N1—H1B	0.8900	C11—H11A	0.9700
N1—H1C	0.8900	C11—H11B	0.9700
O2—C11	1.417 (3)	C12—H12A	0.9700
O2—C10	1.431 (3)	C12—H12B	0.9700
O1—C12^i^	1.428 (3)	C17—C18^i^	1.386 (3)
O1—C12	1.428 (3)	C17—C18	1.386 (3)
C13—C14	1.370 (2)	C17—C19	1.511 (4)
C13—C14^i^	1.370 (2)	C18—H18	0.9300
C9—C10	1.485 (3)	C19—H19A	0.9600
C9—H9A	0.9700	C19—H19B	0.9600
C9—H9B	0.9700	C19—H19C	0.9600
			
O10—Cl1—O10^i^	110.61 (17)	O4—C7—H7A	110.0
O10—Cl1—O8	109.50 (10)	C8—C7—H7A	110.0
O10^i^—Cl1—O8	109.50 (10)	O4—C7—H7B	110.0
O10—Cl1—O9	109.54 (9)	C8—C7—H7B	110.0
O10^i^—Cl1—O9	109.54 (9)	H7A—C7—H7B	108.4
O8—Cl1—O9	108.11 (15)	O2—C10—C9	108.74 (17)
C8—O3—C9	111.86 (15)	O2—C10—H10A	109.9
C7^i^—O4—C7	112.5 (2)	C9—C10—H10A	109.9
C13—N1—H1A	109.5	O2—C10—H10B	109.9
C13—N1—H1B	109.5	C9—C10—H10B	109.9
H1A—N1—H1B	109.5	H10A—C10—H10B	108.3
C13—N1—H1C	109.5	O2—C11—C12	108.77 (19)
H1A—N1—H1C	109.5	O2—C11—H11A	109.9
H1B—N1—H1C	109.5	C12—C11—H11A	109.9
C11—O2—C10	112.44 (17)	O2—C11—H11B	109.9
C12^i^—O1—C12	112.6 (2)	C12—C11—H11B	109.9
C14—C13—C14^i^	120.6 (3)	H11A—C11—H11B	108.3
C14—C13—N1	119.68 (13)	O1—C12—C11	109.48 (17)
C14^i^—C13—N1	119.68 (13)	O1—C12—H12A	109.8
O3—C9—C10	109.56 (17)	C11—C12—H12A	109.8
O3—C9—H9A	109.8	O1—C12—H12B	109.8
C10—C9—H9A	109.8	C11—C12—H12B	109.8
O3—C9—H9B	109.8	H12A—C12—H12B	108.2
C10—C9—H9B	109.8	C18^i^—C17—C18	117.1 (3)
H9A—C9—H9B	108.2	C18^i^—C17—C19	121.45 (14)
O3—C8—C7	109.42 (16)	C18—C17—C19	121.45 (14)
O3—C8—H8A	109.8	C14—C18—C17	121.8 (2)
C7—C8—H8A	109.8	C14—C18—H18	119.1
O3—C8—H8B	109.8	C17—C18—H18	119.1
C7—C8—H8B	109.8	C17—C19—H19A	109.5
H8A—C8—H8B	108.2	C17—C19—H19B	109.5
C13—C14—C18	119.4 (2)	H19A—C19—H19B	109.5
C13—C14—H14	120.3	C17—C19—H19C	109.5
C18—C14—H14	120.3	H19A—C19—H19C	109.5
O4—C7—C8	108.33 (16)	H19B—C19—H19C	109.5

Symmetry codes: (i) *x*, −*y*+1/2, *z*.

### Hydrogen-bond geometry (Å, °)

**Table:**

*D*—H···*A*	*D*—H	H···*A*	*D*···*A*	*D*—H···*A*
N1—H1B···O2^i^	0.89	2.23	2.9477 (19)	138.
N1—H1B···O3^i^	0.89	2.19	2.9511 (18)	143.
N1—H1C···O4	0.89	2.20	2.924 (3)	138.
N1—H1C···O3	0.89	2.20	2.9511 (18)	142.
N1—H1A···O2	0.89	2.22	2.9477 (19)	139.
N1—H1A···O1	0.89	2.15	2.888 (3)	140.

Symmetry codes: (i) *x*, −*y*+1/2, *z*.
